# Childhood mitochondrial encephalomyopathies: clinical course, diagnosis, neuroimaging findings, mtDNA mutations and outcome in six children

**DOI:** 10.1186/1824-7288-39-60

**Published:** 2013-09-26

**Authors:** Jun Lu, Yuanyuan Huang

**Affiliations:** 1 Department of Pediatrics Haikou Municipal People’s Hospital, 43 Renmin Road, Haikou, Hainan Province 570208, P.R. China

**Keywords:** Mitochondrial encephalomyopathy, mtDNA, MELAS, Leigh’s syndrome

## Abstract

Mitochondrial dysfunction manifests in many forms during childhood. There is no effective therapy for the condition; hence symptomatic therapy is the only option. The effect of symptomatic therapy are not well known. We present clinical course, diagnosis and effect of current treatments for six children suffering from mitochondrial encephalomyopathy identified by clinical demonstrations, brain MRI findings and DNA mutations. Two were male and four were female. Their age ranged between 2 and 17 years. Skeletal muscle biopsies were obtained in three and one showed misshaped and enlarged mitochondria under electron microscope. mtDNA mutation frequency was >30%. Five children were diagnosed with MELAS (mitochondrial encephalopathy, lactic acidosis, and strokelike episodes) and one with Leigh’s syndrome (LS). All were given cocktail and symptomatic treatments. One of the five MELAS children died from severe complications. The other four MELAS children remain alive; four showed improvement, and one remained unresponsive. Of the four who showed improvement, two do not have any abnormal signs and the other two have some degree of motor developmental delay and myotrophy. The LS child is doing well except for ataxia. Until better therapy such as mitochondrial gene therapy is available, cocktail and symptomatic treatments could at least stabilize these children.

## Background

Mitochondrial disorders are a group of metabolic diseases, which may cause any symptom, may present at any age, have harmful effects on any tissue and occur by any inheritance pattern [1]. Mitochondrial dysfunction causes a huge burden of disability. Mitochondrial encephalopathy, lactic acidosis, and stroke-like episodes (MELAS) is a more frequently identified mitochondrial disorder.

Currently, there is no effective remedy for mitochondrial disorders. Potential therapies such as stem cell transplantation and gene therapy are only in preliminary stages, making cocktail (*i.e.* supplementation with carnitine, coenzyme Q10, thiamine, L-arginine, folate, *etc.*) and symptomatic treatments the only options for clinical treatment. A combination of nutraceutical compounds often referred to as “mitochondrial cocktail” is based upon a rational attempt to target the final common pathways of mitochondrial dysfunction and provision of alternative energy sources [2]. In this context, we present clinical diagnostic aspects, cocktail and symptomatic treatments, efficacy of such treatment and course of mitochondrial encephalomyomathy (ME) in six children.

## Cases

**Figure 1:**
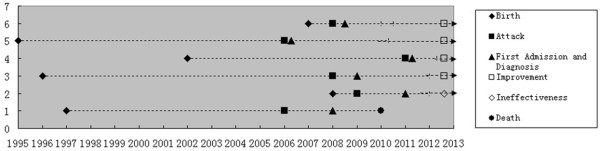
**Clinical courses of six patients.** Note: Vertical axis: patients 1–6, horizontal axis: clinical courses in different calendar years.

Six children were identified with ME at Haikou People’s Hosptial, Hainan, China between October 2006 and October 2012 by clinical demonstrations, brain MRI and blood DNA analysis. Three of the six ME children were subjected to skeletal muscle biopsies. The mtDNA from blood samples was analyzed in all patients. Five children were diagnosed with MELAS and one child with Leigh’s syndrome. All patients were given cocktail and symptomatic treatments, and followed up for one to six years. Cocktail therapy included carnitine (20 mg/kg/d), coenzyme Q10 (5 mg/kg/d), L-arginine (0.1 g/kg/d), adenosine triphosphate (ATP)(80–140 mg/d), vitamin C (0.5-1.0 g/d), monosialotetrahexosylganglioside (40–80 mg/d), folate (1 mg/d), creatine phosphate (1–3 g/d), thiamine (VB1) (25 mg/d), riboflavin (VB2) (25 mg/d) and vitamin B6 (25 mg/d). The treatment was initiated in the hospital and continued at home following discharge. Symptomatic treatments, such as anti-epilepsy, anti-arrhythmia, anti-infection and antilactic acidosis that are important in stabilizing the disease condition were also given when needed. These patients were admitted to hospital when there were severe complications requiring specific treatment, *e.g*. mechanic ventilation support in an intensive care unit for respiratory failure. During follow ups, we observed these patients’ clinical course (Figure 1) brain images, effect of treatment and outcome. The outcome was mainly based on three aspects: improvement, ineffectiveness, and death.

Table 1 shows clinical characteristics and laboratory findings of six ME children. The mean age of onset of ME was 7.2 ± 4.5 years (range, 1-12 years). The mean age at first hospital admission was 8.0 ± 4.4 years (range, 1–13 years). Two children were male and four were female. All patients had symptoms and signs indicative of mitochondrial disorders on admission, mostly demonstrating neuromuscular disorders. Most of the patients had neuromuscular and sensory organ disorders (including seizures, motor abnormality, deafness, eye problem, *etc.*) either at onset or on admission, two patients had gastrointestinal disorders and one had signs of cardiac arrhythmia. The serum lactic acid levels were markedly elevated in all six patients indicating severe mitochondrial involvement. The serum creatine kinase (CK) levels were also abnormally high suggesting the involvement of mostly the muscles that are rich in mitochondria. In brain MRI abnormal signals were often seen in occipital, parietal and temporal lobes in MELAS and the sites of these signals changed with time. The changing pattern from restricted involvement of bilateral cerebral peduncle and brainstem tegmental areas to involvement of basal ganglia area was characteristically seen in patients with Leigh’s syndrome. On electroscope findings of skeletal muscle biopsies, one patient (patient 1) had abnormal mitochondria that is typical of a mitochondrial disorder; two others (patient 3 and patient 4) demonstrated atrophic myopathies with no obvious variations of mitochondria.

**Table 1:** Clinical characteristics and laboratory findings on admission

**Patients**		**At the time of disease onset**	**On admission**				
	**Age (years)**	**Clinical feature**	**Age (years)**	**Clinical features**	**Laboratory finding (serum)**		
					**Lactic acid (mmol/L) (<2.0 normal)**	**Creatine kinase (IU/L) (22 ~ 270 normal)**	**Brain MRI findings**
1	9	Twitching,stroke like episode	11	Deafness, twitching, glossolalia, mobility limitation	6.7	118	Patchy T1 and T2 abnormal signals in right temporal, occipital and parietal lobes and left parietal lobes.
2	1	Mobility limitation	3	Mobility and speaking limitation, amyotrophy, monophasia	4.58	276	Spotted T1 and T2 abnormal signals in left corona radiata.
3	12	Vomiting,headache, diarrhea	13	Vomiting, lethargy, hypophrenia, speaking and mobility limitation	4.5	602	Multiple patchy T1 and T2 abnormal signals in bilateral cerebellar hemisphere, both occipital lobes and right parietal lobe.
4	9	Vomiting, headache, lethargy	9	Vomiting, headache, lethargy, positive reflex	2.8	766	Large patchy T1 and T2 abnormal signals in left temporo-occipito-parietal lobes.
5	11	Pitting edema, tachycardia, blurred vision, seizures	11	Pitting edema, tachycardia, blurred vision, seizures	3.0	277	Large patchy T1 and T2 abnormal signals in bilateral temporo-occipito-parietal lobes.
6	1	Progressive motor retardation, tremor, ptosis	1	Progressive motor retardation, tremor, ptosis	3.9	138	Abnormal signals in bilateral cerebral peduncle and brainstem tegmental area.

Note: The mean level of lactic acid was 4.25 ± 1.41 mmol/L, the mean level of creatine kinase was 362.83 ± 262.73 IU/L. These brain MRI findings represent those at admission, subsequent brain MRI during follow up showed different degree of changes in different sites.

Table 2 shows mtDNA mutations in patients and their mothers. All six patients had a DNA mutation frequency of more than 30%. The mean was 48.2% ± 15.5%. The carrier mother (with no clinical manifestation) of patient 1 and patient 2 with MELAS had a low mutation frequency of 3.8% and the carrier mother of patient 6 with LS had a higher mutation frequency of 30%.

**Table 2:** Mitochondrial DNA mutations from blood samples

**Patients**	**Clinical phenotype**	**DNA mutations**	**Mutation frequency**	**Patient’s mother’s mutation frequency**
1	MELAS	m.3243A>G	34%	3.8%
2	MELAS	m.3243A>G	53%	3.8%
3	MELAS	m.3243A>G	47.4%	N
4	MELAS	m.3243A>G	47.6%	N
5	MELAS	m.3243A>G	32.3%	N
6	Leigh’s Syndrome	m.13513G>A	75%	30%

MELAS = mitochondrial encephalopathy, lactic acidosis, and stroke-like episodes.

**Figure 2:**
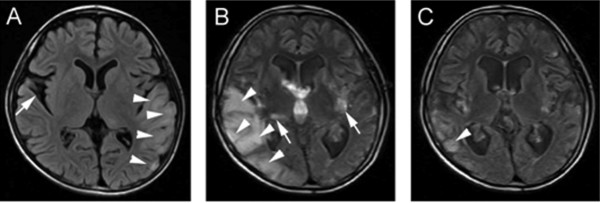
**Brain MRI (FLAIR, fluid-attenuated inversion recovery) of patient 1: (A) taken one year before admission;: Abnormal signals in the left occipital, parietal, and temporal lobes (arrowheads) and a cyst (arrow) in the right parietal area. (B)** Abnormal MRI signals shifted to the right side (arrowheads) at admission from previous left side. Symmetrical lesions at the lenticular nuclei (arrow) were also observed. **(C)** These abnormalities were dramatically decreased (arrowheads) after 30 days cocktail treatment in hospital.

**Figure 3:**
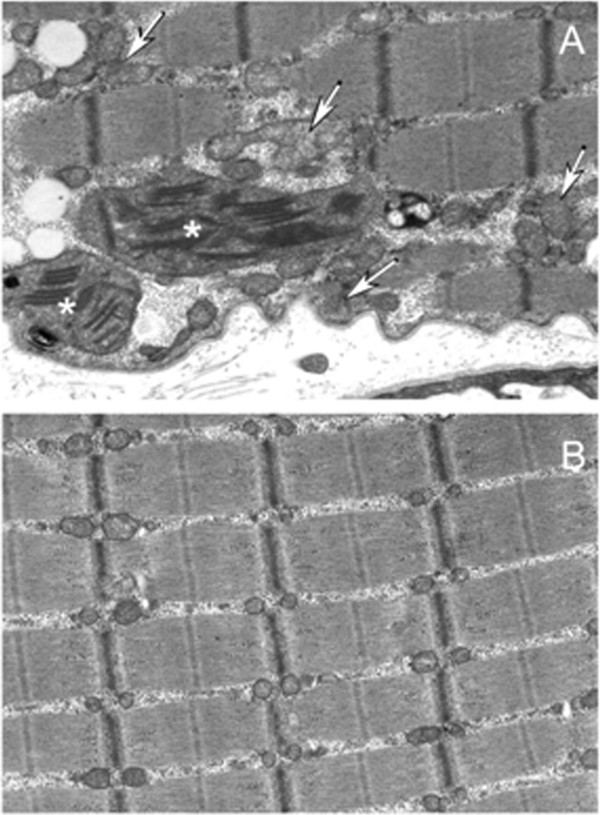
**Electron microscopy examination of the biceps brachii muscle: (A) Biopsy from patient 1 on admission shows misshaped mitochondria (arrows), and enlarged mitochondria with inclusion bodies (asterix). (B)** Biopsy from a control subject.

**Figure 4:**
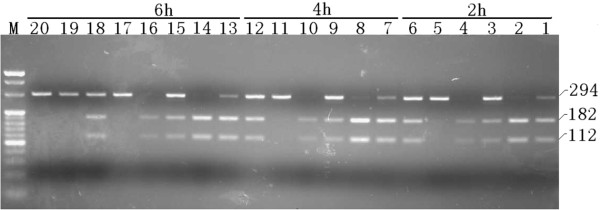
**Mutations in family member by restriction fragment length polymorphism analysis using a 294-base pair fragment, amplified by polymerase chain reaction (PCR).** The figure shows the digest result of blood and urine samples of patient 1 and 2, their mother and the negative control. After digestion of the target gene, two fragments (182 bp and 112 bp) were generated, using a blood and a urine samples from three members of the family (3, 9, 15 were blood samples and 4,10,16 were urine samples of patient 1; 1, 7, 13 were blood samples and 2, 8, 14 were urine samples of patient 2; 5, 11, 17 were blood samples and 6, 12,18 were urine samples of mother; 19,20 were controls). The figure confirms the A3243G mutation point in amplified products of these samples. The gene in the mother’s blood sample was almost not digested (with a mutation frequency of only 3.8%). The gene in the negative control (i.e. that does not contain this mutation) was not digested.

Special features of two siblings (patient 1 and patient 2):

Patient 1 and patient 2 are siblings, they were both diagnosed with MELAS, and their mothers are carriers. The diagnosis was based on the clinical manifestations, laboratory findings (Table 1), typical brain MRI findings(patient 1, Table 1 and Figure 2), biceps brachii biopsy findings (patient 1 and Figure 3), and family mtDNA mutations (Figure 4). The two siblings were given cocktail and symptomatic treatments for two years after diagnosis. However, patient 1 died after two years of treatment because of severe complications of massive gastrointestinal haemorrhage and shock following recurrence of a stroke-like episode. Patient 2 was stabilized after one year of treatment, but she still could not speak, and had amyotrophy and severe mobility limitations.

Figure 1 shows clinical courses of mitochondrial diseases in six patients. Three patients were admitted to hospital at the onset of the disease, while the other three admitted with some time delay (about one to two years after disease onset). Until the latest follow up in October 2012, five patients were stabilized with cocktail and symptomatic treatments and, one was free from all symptoms. One patient died at home due to recurrence of a stroke-like episode with severe complications.

## Discussion

Mitochondrion is a vital organelle and its dysfunction can cause various diseases at various levels in cells, tissues, organs and systems. The final result of mitochondrial diseases is the failure for the cell to produce energy in the form of adenosine triphosphate (ATP) leading to multisystem dysfunction [3]. These diseases start in childhood and progress subsequently with significant suffering that result in heavy burdens on affected families. Mitochondrial encephalomyopathy is one of the most frequent clinical phenotype of childhood mitochondrial disorders [4]. For several decades, cocktail and symptomatic treatments remain the only seemingly effective therapy [5], but the outcome of mitochondrial disease with these treatments is unknown.

Mitochondrial diseases occur due to mtDNA mutations or deletions. Other mitochondrial diseases due to mitochondrial structural defect, nDNA mutations, intergenomic signaling defects between nDNA and mtDNA, *etc.* are not easily diagnosed [6]. For many years the most frequently reported mitochondrial disease has been MELAS [7].

Early diagnosis of mitochondrial diseases and effective treatment is a challenge in the medical world. A careful clinical examination and search for typical symptoms [8] is the key that leads to initial diagnostic work-up. Because of highly variable phenotypes of mitochondrial diseases, they are often undiagnosed or misdiagnosed as other neuromuscular diseases, developmental delay, encephalopathy, *etc.* Six patients we described above also had presentations easily leading to misdiagnosis such as vomiting, neuromuscular dysfunction, epilepsy, ataxia, speech delay and ocular disorders. They had a mean time delay for diagnosis of 1 (1.1 ± 0.8) year.

The neuroimaging often give early clues for diagnosis of ME. The brain MRI findings of above six patients showed characteristic abnormal signals in occipital, temporal, parietal lobes and basal ganglia. Typical “protean” manifestations with time were also characteristically observed. Skeletal muscle biopsy and mutational studies help in the diagnosis in the absence of straightforward radiological findings. Observation of mitochondrial ultrastructure in a muscle under electron microscope is useful although all patients may not show the changes [8]. In the present case series one of the three muscle biopsied patients showed misshaped and enlarged mitochondria indicative of mitochondrial dysfunction. The clinical phenotype, MELAS in the above five cases corresponded with m.3243A > G mutation with a mutation frequency of 32.3% to 53% (mean value, 42.9% ± 9.2%). Leigh’ syndrome had m.13513G > A mutation with a mutation frequency of 75%. This aspect shows that there is no direct relationship between the clinical severity and the mutation frequency. The threshold mutation rate of MELAS usually reported in the literature is >70-80% [9]. Quantitative analyses of the mutation among siblings (Figure 4) showed different mutation loads in blood and urine [in patient 1: 34% and 59.7% respectively and in patient 2: 53% and 75.3% respectively]. The mutation loads in blood is lower than in urine. Usually the blood specimen is used to study mutation frequency instead of urine because of the more complicated processing of the urine sample.

The serum biomarkers, such as lactic acid, ammonia, creatine, and glucose are useful to some extent though they lack specificity. The high levels of lactic acid and creatine in all six patients reflected metabolic disorder and muscle damage.

Currently, cocktail therapy enhancing respiratory chain function [9] and symptomatic therapy [4] remain the only treatment options for mitochondrial disorders. We achieved a stabilization rate of 83.3% (5/6) with these treatment options.

Symptomatic treatment focused on the following symptoms: lactic acidosis, epilepsy, respiratory failure, cardiac arrhythmia, malnutrition and abnormalities of muscle tone. We used sodium bicarbonate to treat lactic acidosis when the patient had recurrent stroke-like episodes and presented with shock. To treat epilepsy, we used carbamazepine or diazepam instead of sodium valproate, which has been associated with liver disease. Invasive or non-invasive mechanic ventilation was used for respiratory failure. Regular anti-arrhythmia regimen will delay progression of cardiomyopathy and heart failure. Because of bulbar or pseudobulbar complications of mitochondrial disease, we used nasogastric tube feeding in small children with dysphagia and increased risk of aspiration pneumonia. We cooperated with physiotherapists to treat the patients with dystonia and spasticity, which gave some improvement.

## Conclusion

In conclusion, from our clinical study, we believe cocktail and symptomatic treatments, at present, can stabilize childhood mitochondrial diseases to some extent. The long term benefits of these treatment modalities however need to be explored further.

## Consent

Written informed consent was obtained from the parents of these patients for publication of these cases and accompanying images.

## Competing interests

We declare that we have no competing interests.
